# Inhibition of neddylation facilitates cell migration through enhanced phosphorylation of caveolin-1 in PC3 and U373MG cells

**DOI:** 10.1186/s12885-017-3942-9

**Published:** 2018-01-05

**Authors:** Sung Yeon Park, Jong-Wan Park, Gun-Woo Lee, Lan Li, Yang-Sook Chun

**Affiliations:** 1 Ischemic/Hypoxic Disease Institute, Seoul National University College of Medicine, Seoul, 110-799 Republic of Korea; 2 Department of Biomedical Sciences, Seoul National University College of Medicine, Seoul, 110-799 Republic of Korea; 3 Department of Physiology, Seoul National University College of Medicine, Seoul, 110-799 Republic of Korea

**Keywords:** Caveolin-1, MLN4924, Neddylation, Phosphorylation, Cell migration

## Abstract

**Background:**

Protein neddylation is a post-translational modification by a covalent conjugation with the neural precursor cell expressed, developmentally downregulated 8 (NEDD8). Although this process has been reported to participate in diverse cellular signaling, little is known about its role in cancer cell migration. Given a recent proteomics report showing that NEDD8 is downregulated in prostate cancer tissues versus normal prostate tissues, we tested the possibility that neddylation plays a role in cancer evolution, and then tried to identify target proteins of the neddylation.

**Methods:**

The neddylation process was inhibited by transfecting cancer cells with NEDD8-targeting siRNAs or by treating the cells with a NAE1 inhibitor MLN4924. Cell migration was evaluated by an in vitro wound-healing assay and a Transwell migration assay. His/NEDD8-conjugated proteins were pulled down with nickel-affinity beads under a denaturing condition, and identified by Western blotting. All data were processed using the Microsoft Excel program and analyzed statistically by two-sided, unpaired Student’s *t-*test.

**Results:**

Caveolin-1, which plays a critical role in cell migration, was identified to be conjugated with NEDD8. When the neddylation was inhibited, the phosphorylation of caveolin-1 at Tyr14 was augmented in PC3 and U373MG cells, thereby leading to increased cell migration. Such consequences by neddylation inhibition were abolished in the presence of a Src family kinase inhibitor PP2.

**Conclusions:**

NEDD8 seems to inhibit the Src-mediated phosphorylation of caveolin-1 by modifying the structure of caveolin-1 protein, which blocks the migration of cancer cells. Although the neddylation process is currently regarded as an emerging target for cancer therapy, our results suggest the possibility that the inhibition of neddylation could facilitate cancer invasion or metastasis at least in some types of cancers.

**Electronic supplementary material:**

The online version of this article (doi: 10.1186/s12885-017-3942-9) contains supplementary material, which is available to authorized users.

## Background

Protein neddylation is a post-translational modification by a covalent binding of NEDD8 (neural precursor cell expressed developmentally downregulated 8) to proteins. Like the ubiquitination process, neddylation is proceeded in three sequential steps of E1–3. The first step is govern by the NEDD8-activating enzyme (NAE), which is composed of amyloid beta precursor protein-binding protein 1 (APPBP1) and ubiquitin-like modifier activating enzyme 3 (UBA3). The second and third steps are carried out by the NEDD8-conjugating enzyme (UBC12) and variable substrate-specific NEDD8-E3 ligases, respectively [1, 2]. This results in the changes in protein stability and functionality. In case of transcription factors, the neddylation regulates gene expressions by modulating the transcriptional activities of its targets [3–5].

Caveolin-1 is an integral membrane protein implicated in a wide variety of physiological functions [6]. In cancer development, caveolin-1 has been reported to behave as a tumor suppressor in the early stage [7]. By contrast, it is regarded as a tumor promoter because caveolin-1 overexpression facilitates cancer cell migration, invasion and metastasis, and also induces multiple resistances to anticancer agents [8–11]. The *CAV1* gene is presented as two variants through alternative mRNA splicing, Cav1α and Cav1β [12]. Of them, only Cav1α possesses the tyrosine-14 (Y14) residue that is phosphorylated by non-receptor tyrosine kinases, including the proto-oncogene Src [13]. The phosphorylation at Y14 has been reported to promote the localization and stabilization of focal adhesion kinase which is essential for cell migration [14, 15]. Accordingly, the Src-caveolin-1 pathway is believed to be critically involved in cancer cell migration.

Given a recent proteomics report showing that NEDD8 is downregulated in prostate cancer tissues versus normal prostate tissues [16], the authors were encouraged to investigate the role of NEDD8 in prostate cancer promotion. Mechanistically, caveolin-1 was post-translationally modified by NEDD8 conjugation, which attenuated the Src-mediated phosphorylation of caveolin-1 at Y14. Consequently, the inhibition of caveolin-1 neddylation stimulated the migration of prostate cancer and glioblastoma cells. From these results, it is proposed that the neddylation of caveolin-1 stops cell migration at least in prostate cancer and glioblastoma by counteracting the Src-caveolin-1 pathway.

## Methods

### Antibodies and reagents

Antibodies against NEDD8 and Myc tag (Cell Signaling Technology, Danvers, MA), FLAG tag (Sigma-Aldrich, St. Louis, MO), caveolin-1 and Y14-phospho-caveolin-1 (BD Biosciences, San Jose, CA), β-Tubulin, SUMO-1, and ubiquitin (Santa Cruz Biotechnology, Dallas, TX, USA) were purchased from the indicated companies. 4-amino-5-(4-chlorophenyl)-7-(dimethylethyl) pyrazolo [3,4-d] pyrimidine (PP2) was purchased from Calbiochem (San Diego, CA). MLN4924 was synthesized, as described previously [17].

### Cell culture

HEK293T, PC3, U373MG, and A549 cell lines were purchased from the Korean Cell Line Bank (Seoul, Korea). HEK293T was maintained in DMEM. U373MG, PC3, A549 cells in RPMI. All media were supplemented with 10% fetal bovine serum (FBS).

### Western blotting

Total cell lysates were prepared using 2× denaturing SDS sample buffer, subjected to SDS-PAGE, and transferred to an Immobilon-P membrane (Millipore, Bedford, MA). Membranes were blocked with 5% skim milk in TTBS for 1 h and then were incubated overnight at 4 °C with the primary antibody. Membranes were incubated with a horseradish peroxidase-conjugated secondary antibody for 1 h at room temperature, and stained with the enhanced chemiluminescent-plus reagent (Thermo Fisher Scientific, Rockford, IL).

### Transient transfection

For transient transfection, cells were transfected with siRNAs using Lipofectamine® RNAiMax™ (Invitrogen, Carlsbad, CA) or with plasmids using the calcium phosphate reagent. Transfected cells were stabilized for 48 h before subsequent experiments. The siRNA duplexes were synthesized by Integrated DNA Technologies (Hanam, South Korea), and their nucleotide sequences are as follows:

- caveolin-1^#1^, 5′-CCUUCACUGUGACGAAAUACUGGTT-3′;
- caveolin-1^#2^, 5’-GCAGUUGUACCAUGCAUUAAGAGCT-3′;
- NEDD8^#1^, 5′-UCCUUGAUUCGCUCCACCUUGUCUGUG-3′;
- NEDD8^#2^, 5’-UUCACUUUAAUUAGCAUCUUCUUCCCA-3′.

FLAG- and His-tagged plasmids was constructed as described previously [3], and GFP-tagged caveolin-1 was kindly given by Dr. Sang Jeong Kim (Seoul National University, Seoul, South Korea) and Myc-tagged caveolin-1 was constructed by replacing GFP with myc tagging. GFP-tagged caveolin-1-K5R was generated by site directed mutagenesis.

### Identification of NEDD8 conjugation

Identification of NEDD8 conjugation was performed and modified based on the description in Jaffray and Hay [18]. After transfected with His-tagged NEDD8 or NEDD8ΔGG plasmid, cells were lysed in a denaturing buffer (6 M guanidine hydrochloride, 0.1 M Na_2_HPO_4_/NaH_2_PO_4_, 0.01 M Tris-HCl, pH 8.0, plus 10 mM imidazole and 10 mM β-mercaptoethanol). The lysates were mixed with Ni^2+^-NTA agarose beads (Qiagen, Valencia, CA) and incubated for 4 h at room temperature using a rotator. The beads were successively washed for 5 min each with the following solutions: lysis buffer (pH 8.0), washing buffer (pH 8.0; 8 M urea, 0.1 M Na_2_HPO_4_/NaH_2_PO_4_, 0.01 M Tris-HCl, pH 8.0, plus 20 mM imidazole, and 10 mM β-mercaptoethanol), washing buffer (pH 6.3) plus 0.2% Triton X-100, and washing buffer (pH 6.3) plus 0.1% Triton X-100. Then, the beads were eluted with SDS sample buffer and analyzed by Western blotting.

### Immunoprecipitation

For immunoprecipitation, cell lysates (1 mg of protein) were incubated with 5 μL of antibody for 2 h and then incubated with 10 μL of protein A/G-Sepharose® beads (GE Healthcare, Pittsburgh, PA) for 4 h at 4 °C. After washing, the immunoprecipitated proteins were eluted in SDS sample buffer and subjected to SDS-PAGE and Western blotting.

### Wound healing assay

Cultured cells were grown in 12-well plates until they reached confluence. The medium was removed and the cells were washed with PBS three times before culturing was continued in serum-free medium for an additional 24 h. Then, a rectangular lesion was created in the monolayers with a pipette tip. Cells were washed at least three times with PBS to remove debris and then cultured in serum-free medium. After 24 h, three randomly selected fields at the lesion border were assessed under an inverted microscope. The area of migration was measured using the ImageJ software (National Institutes of Health, Bethesda, MD).

### Transwell migration assay

Assays were performed in Boyden chambers (Transwell® Costar®; 6.5 mm diameter, 8 μm pore size) according to the manufacturer’s protocol. Briefly, the bottom sides of the inserts were coated with 0.5 mg/mL collagen. Cells (2.5–5 × 10^4^), re-suspended in 100 μL serum-free medium containing the designated concentration of reagents, were plated in the top of each chamber insert and the bottom chambers were filled with 600 μL complete medium containing 10% FBS. Cells were allowed to migrate for 24 h. Stationary cells on the top surface of the inserts were scraped with a cotton swab, and the cells that migrated to the bottom side of the inserts were fixed with methanol, washed, and stained with 0.1% crystal violet in 2% methanol. Images were acquired using an inverted microscope, and the number of cells that migrated to four independent areas per filter was counted using the ImageJ software.

### Statistical analysis

All data were analyzed using Microsoft Excel 2007 and expressed as the means and standard deviations (*sd*). Continuous variables were analyzed using Student’s *t-*tests if the data were normally distributed. All statistical tests were two-sided. *P* values <0.05 were considered to indicate statistical significance.

## Results

### Neddylation inhibition augments cell migration in PC3 and U373MG

**Fig. 1:**
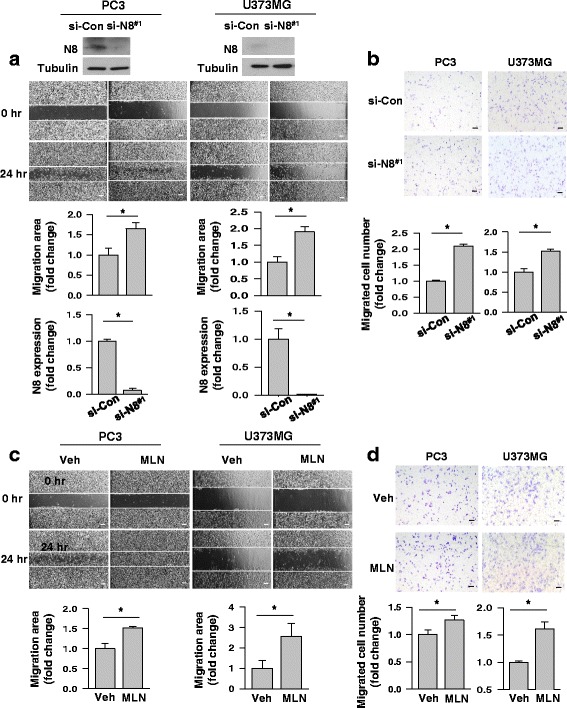
Migration of some cell-lines was augmented by neddylation block. **a** PC3 and U373MG cells, which had been transfected with NEDD8 (N8)-targeting (si-NEDD8 #1) or non-targeting (si-Con) siRNAs, were lysed and subjected to Western blotting with antibodies to NEDD8 and β-tubulin (top). The migration potential of the transfected cells were evaluated for 24 h using the scratch-based wound healing analysis. The area of cell migration was calculated using the ImageJ software (middle). The efficiency of the NEDD8 knock down was quantified based upon the relative level of β-tubulin (bottom). **b** Transwell migration assay was performed to evaluate migration potential of cells. PC3 or U373MG cells, which had been transfected as described in the A panel, were loaded in the upper chamber. After 24 h-incubation, the lower surface of the interface membrane was pictured (top). Cells on the membrane were counted (bottom). Results (means + SDs, *n* = 3) are presented as relative values vs. the si-Con group. *P < 0.001 vs the si-Con. **c** Scratch-based wound healing was performed on PC3 and U373MG cells in the presence of MLN4924 (0.25 μM and 0.5 μM) or DMSO as a control. Cell migration was evaluated for 24 h using the scratch-based wound healing analysis (top) and the migration area was calculated using ImageJ (bottom). **d** Transwell migration assay was performed on PC3 and U373MG cells in the presence of MLN4924 (0.25μM and 0.5μM) or DMSO as a control. After 24 h-incubation, Cells on the lower surface of the membrane were pictured (top) and counted (bottom). All results are presented as the means + standard deviation of three independent experiments, and * denotes *P* < 0.05 between the indicated groups. Scale bar = 200 μm

Given a recent proteomics report showing that NEDD8 is downregulated in prostate cancer tissues versus normal prostate tissues [16], we examined whether the downregulation of NEDD8 is associated with cell migration in a prostate cancer cell-line PC3 and in a glioblastoma cell-line U373MG. The NEDD8 down-regulation was achieved using a siRNA targeting NEDD8 (Fig. 1a, top). Interestingly, NEDD8-deficient PC3 and U373MG cells were found to migrate more to the scratched, cell-free zone than control cells (Fig. 1a, middle and bottom). We confirmed the negative role of NEDD8 in cell migration using the Transwell system (Fig. 1b). Next, we inhibited the neddylation process using a NAE inhibitor MLN4924 in the range of 0.25–0.5 μM, in which it selectively inhibits NEDD8 activating enzyme (NAE) without influencing the related enzymes ubiquitin-activating enzyme (UAE) and SUMO-activating enzyme (SAE) (Additional file 1) [19]. In both the wound healing (Fig. 1c) and the Transwell migration analyses (Fig. 1d), the migration of PC3 and U373MG cells were shown to be significantly enhanced by MLN4924. These results suggest that cell migration is negatively controlled through the neddylation process.

### Caveolin-1 is neddylated and then dephosphorylated in prostate and glioblastoma cells

**Fig. 2:**
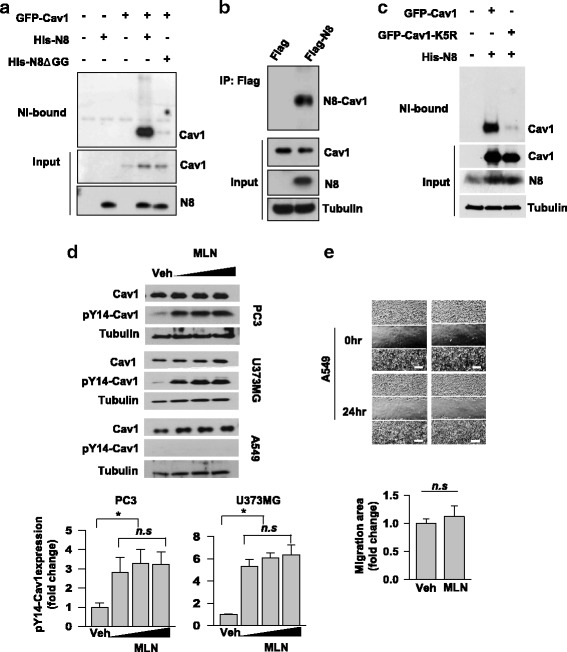
Neddylation antagonized the phosphorylation of caveolin-1 at Y14. **a** Ni-NTA-binding assay was performed in HEK293T cells co-expressing GFP-caveolin-1 (Cav1) and His-NEDD8 (N8) or His-NEDD8ΔGG (N8ΔGG). Neddylated proteins were pulled down with Ni-NTA beads under a denaturing condition, and subjected to Western blotting with the indicated antibodies. **b** HEK293T cells expressing FLAG tag or FLAG-NEDD8 were lysed and subjected to co-immunoprecipitation analysis. Precipitated and input proteins were analyzed by Western blotting. **c** Ni-NTA-binding assay was performed in HEK293T cells co-expressing His-NEDD8 and GFP-caveolin-1 or GFP-caveolin-1-K5R. Neddylated proteins were pulled down with Ni-NTA beads under a denaturing condition, and subjected to Western blotting with the indicated antibodies. **d** PC3, U373MG, and A549 cells were treated with MLN4924 (0.125, 0.25, or 0.5 μM) or DMSO (Veh) for 24 h and then subjected to Western blotting (top). pY14-Cav1 represents caveolin-1 phosphorylated at Y14 residue. The level of pY14-Cav1 along with the range of MLN4924 was quantified based upon the relative level of β-tubulin from PC3 and U373MG cell lysates (bottom). **e** A549 cells were treated with 0.5 μM MLN4924 and subjected to the wound-healing analysis for 24 h. Each bar represents the means + standard deviation of migration areas from three independent experiments, and n, s, denotes *P* > 0.05 between the indicated groups. Scale bar = 200 μm

Given in vitro and in vivo studies previously showing that caveolin-1 overexpression enhanced cancer invasion and metastasis (9, 10), we tested the possibility that caveolin-1 is involved in the migration inhibition by neddylation. For this purpose, HEK293T cells were co-transfected with C terminal GFP tagged-caveolin-1 and His-NEDD8 or His-NEDD8ΔGG (a conjugation-defective mutant), and neddylated proteins were pulled down under a denaturing condition using Ni^2+^-affinity beads. Caveolin-1 was identified to be covalently conjugated with NEDD8, but not with NEDD8ΔGG (Fig. 2a). Furthermore, an immunoprecipitation assay demonstrated that endogenous caveolin-1 was also conjugated with expressed FLAG-NEDD8 (Fig. 2b). The neddylation of caveolin-1 was for the first time identified in this experiment. Furthermore, in the process of identifying the amino acid in caveolin-1 responsible for conjugating to NEDD8, we assumed that one of the lysine residues in N terminal is essential for neddylation, since N terminal myc-tagged caveolin-1 fails to be conjugated to NEDD8, while C terminal GFP-tagged caveolin-1 was conjugated to NEDD8 (Additional file 2 and Fig. 2a). Myc tag may interfere nearby lysine residues conjugating to NEDD8 by masking some portion of N terminal. Then, we examined lysine residues in N terminal using site directed mutagenesis. Fortunately, we identified that K5, the first lysine among the N terminal, is responsible for conjugating to NEDD8 due to the fact that the neddylation was almost abolished with caveolin-1-K5R (Fig. 2c). Then, how does NEDD8 regulate caveolin-1? To check the possibility that neddylation determines stability of caveolin-1, we measured the cellular levels of caveolin-1 in prostate, glioblastoma, and lung carcinoma cells which had been treated with MLN4924. However, the amount of caveolin-1 protein was not affected in all tested cells treated with MLN4924 (Fig. 2d). Next, we examined if caveolin-1 is functionally regulated by neddylation. Since the phosphorylation of caveolin-1 at Y14 has been known to be crucial for cell migration [14, 15], we measured the Y14-phosphorylation of caveolin-1 in MLN4924-treated cells. After MLN4924 treatment, the Y14-phosphorylation was markedly increased in PC3 and U373MG cells, but not in lung carcinoma cell line A549 (Fig. 2d). Given that MLN4924 failed to stimulate cell migration in A549 showing no Y14-phosphorylation, the Y14-phosphorylation seems to be required for the MLN4924-induced migration (Fig. 2e).

### Caveolin-1 phosphorylation at Y14 is essential for MLN4924-induced cell migration

**Fig. 3:**
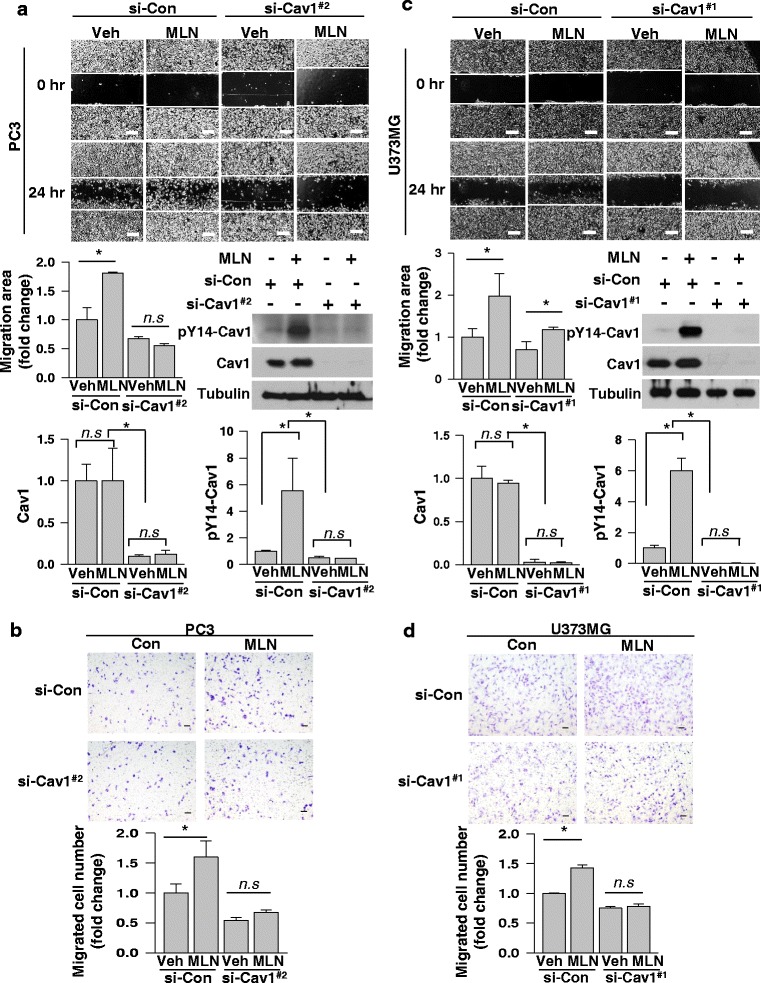
Phosphorylated caveolin-1 is in charge of MLN4924-induced cell migration. **a**, **c** Scratch-based wound healing assays were performed for 24 h in PC3 (**a**) and U373MG (**c**) cells which were depleted of caveolin-1 using siRNAs (#2 and #1, respectively) and si-control in the presence of MLN4924 (0.25 μM and 0.5 μM) or DMSO (top). The migration areas were calculated using ImageJ (middle, left). Proteins in cells lysates were analyzed by Western blotting (middle, right). The efficiency of the Caveolin-1 knockdown and magnitude of the phosphorylation of Caveolin-1 was quantified based upon the relative level of β-tubulin (bottom). **b**, **d** Transwell migration assays were performed in PC3 (**b**) and U373MG (**d**) cells which were depleted of caveolin-1 using siRNAs (#2 and #1, respectively) and si-control in the presence of MLN4924 (0.25 μM and 0.5μM) or DMSO for 24 h (top), and migrated cells were counted (bottom). Each bar represents the means + standard deviation of results from three independent experiments. * denotes *P* < 0.05 and n, s, does *P* > 0.05 between the indicated groups. Scale bar = 200 μm

To further examine the involvement of caveolin-1 in cell migration, cell migration was evaluated in PC3 and U373MG cells where caveolin-1 was knocked down. Cell migration was double-checked using wound-healing and Transwell migration analyses. In both assays, the basal migration of PC3 cells was somewhat decreased by caveolin-1 knock-down. More importantly, MLN4924 failed to stimulate cell migration under caveolin-1 knock-down (Fig. 3a and b). Likewise, the basal migration of U373MG cells was attenuated by caveolin-1 knock-down and the enhanced migration by MLN4924 was decreased under caveolin-1 knock-down (Fig. 3c and d). Additionally, caveolin-1 knock-down with different kinds of siRNAs have shown similar effects in PC3 and U373MG cells (Additional file 3). These results strongly indicate that MLN4924 enhances cell migration via the activation of caveolin-1.

### Neddylation inhibition stimulates caveolin-1 phosphorylation through the Src kinase

**Fig. 4:**
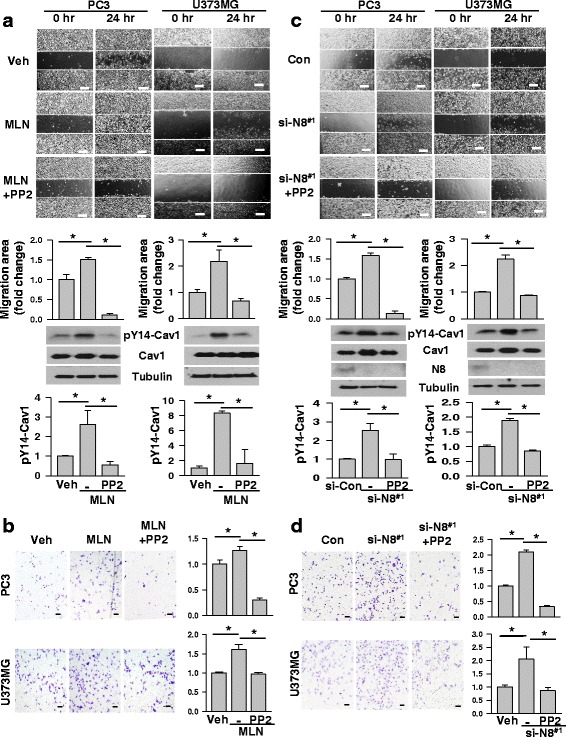
Neddylation inhibition enhances the Src-mediated phosphorylation of caveolin-1. **a** Scratch-based wound healing assays were performed for 24 h in PC3 and U373MG cells which were treated with MLN4924 (0.25 μM and 0.5 μM) or/and 10 μM PP2 (top). The migration areas were calculated using ImageJ at just below. Protein levels in cells lysates were analyzed by Western blotting (middle). The PP2 mediated inhibition of the phosphorylation of Caveolin-1 was quantified based upon the relative level of β-tubulin (bottom). **b** Transwell migration assays were performed in cells treated with MLN4924 (0.25 μM and 0.5 μM) or/and 10 μM PP2 (left), and migrated cells were counted (right). **c** Both PC3 and U373MG cells, which had been depleted of NEDD8 using siRNA #1 and si-control, were subjected to wound healing assay for 24 h in the absence or presence 10 μM PP2 (top). The migration areas were calculated using ImageJ at just below. Protein levels in cells lysates were analyzed by Western blotting (middle). The PP2 mediated inhibition of the phosphorylation of Caveolin-1 was quantified based upon the relative level of β-tubulin (bottom). **d** Transwell migration assays were performed in cells which were prepared as described in C panel (left), and migrated cells were counted (right). Each bar represents the means + standard deviation of results from three independent experiments. * denotes *P* < 0.05 between the indicated groups. Scale bar = 200 μm

It has been reported that the Src kinase-mediated phosphorylation of caveolin-1 at Y14 facilitates cell migration [15, 20]. Thus, we examined the involvement of Src in cell migration stimulated by neddylation block. In both PC3 and U373MG cells, a Src inhibitor PP2 successfully prevented the caveolin-1 phosphorylation by MLN4924, and also almost completely abolished the cell migration-stimulating effect of MLN4924 (Fig. 4a and b). Likewise, PP2 attenuated the caveolin-1 phosphorylation and cell migration stimulated by NEDD8 knock-down (Fig. 4c and d). These results support our notion that the neddylation of caveolin-1 controls cell migration in prostate cancer and glioblastoma cells by deregulating the Src-dependent phosphorylation of caveolin-1. Additionally, NEDD8 knock-down with different kind of siRNAs has shown similar effects in U373MG cells. However, compared to NEDD8 knock-down with siRNA #1, PC3 cells showed less effect in migration probably due to less augmentation in phosphorylation of caveolin-1 under NEDD8 knock-down with siRNA #2. The migration and the caveolin-1 phosphorylation in both PC3 and U373MG cells were attenuated by PP2 treatment (Additional file 4). Interestingly, basal migration of the vehicle and si-control samples in both PC3 and U373MG cells was attenuated by the treatment of PP2. Cell migration was double-checked using wound-healing and Transwell migration analyses (Additional file 5). Probably, PP2 can inhibit other protein kinases involving migration mediated through dephosphorylation of proteins as well as caveolin-1 [21].

## Discussion

In this study, NEDD8 knock-down enhanced cell migration in PC3 and U373MG cells. As caveolin-1 was identified to be conjugated with NEDD8, we hypothesized that the neddylation of caveolin-1 determines cancer cell migration. Although it was not regulated in the protein level by neddylation, caveolin-1 was functionally regulated by neddylation. Caveolin-1 is activated by being phosphorylated at the Y14 residue, which was found to be inhibited by the neddylation of caveolin-1. The inhibition of neddylation, which was achieved using NEDD8-targeting siRNAs or MLN4924, enhanced the Src-mediated Y14-phosphorylation of caveolin-1, thereby promoting the migration of PC3 and U373MG cells.

Several target proteins of neddylation have been identified; the most intensively-characterized substrate is the cullin family, a component of Really Interesting New Gene (RING) E3-ubiquitin ligases (cullin ring ligases, CRLs). Neddylation of cullins stimulates the activity of the ubiquitin E3 ligase, resulting in increased proteasomal degradation of proteins which include tumor suppressors, cell cycle regulators, components of the DNA replication machinery, and mediators of cellular stress [2, 5, 22, 23]. In addition, neddylation has been reported to stabilize hypoxia-inducible factor 1-alpha (HIF-1α) and transforming growth factor-beta receptor II [3, 24]. Neddylation is also involved in regulating the transcriptional activities of several substrates. For example, when cell-cycle-regulating transcription factor E2 is subjected to neddylation, the transcription factor is degraded and functionally repressed, leading to impaired cell growth [25, 26]. The p53 tumor suppressor is another target of neddylation which is mediated by the mouse double minute 2 (Mdm2), and its transcriptional activity is repressed by neddylation [27]. Moreover, neddylation has been shown to regulate cell movement. Renaudin et al. demonstrated that neddylation promotes the trafficking of C-X-C chemokine receptor type 5 to the plasma membrane and by doing so enhances cell migration [28]. We here identified carveolin-1 as a new target of neddylation, and to our best knowledge, this is for the first time reported in this study.

Cell migration is a pivotal biological process required in processes such as embryonic development and tissue repair and regeneration; it is also involved in pathological conditions, including arthritis, atherosclerosis, and the metastasis of cancer cells [29]. This event arises through the dynamic interplay of multiple cellular components associated with cell adhesion and movement. First, the microtubule organizing center is polarized towards the leading edge of the cell [30]. Once oriented, the cell extends in the direction of migration either via broad (lamellipodia) or focused (filopodia) protrusions. Then, interactions with many molecules related to focal contacts and integrins allow the cell to migrate in the desired direction [31–33]. Consistent with this, we observed that the lamellipodia at the edge of U373MG cells were extended more after treatment with MLN4924 or the depletion of NEDD8 compared with the control, which may facilitate dragging for movement (data not shown). In recent years, a strong connection has been established between caveolin-1 and cell migration/invasion. In particular, phosphorylation on Y14 of caveolin-1 appears to be required for cell migration. The caveolin-1 phosphorylation facilitates anchorage-independent growth by recruiting growth factor receptor-bound protein 7 (Grb7) [34], integrin-dependent internalization of membrane micro-domains [35], and activation of matrix metalloproteinases [20]. Additionally, the caveolin-1 phosphorylation is involved in the localization and stabilization of focal adhesion kinase (FAK) essential for cell migration [15]. Considering such roles of phosphorylated caveolin-1, it is not surprising that cancer cell migration is enhanced by inhibiting neddylation.

MLN4924, a first-in-class NAE inhibitor, has shown therapeutic efficacy in preclinical studies [36]. In clinical trials, MLN4924 also showed a modest effect against acute myeloid leukemia [37]. As it inactivates CRLs, MLN4924 accumulates tumor-suppressive CRL substrates, which induces genotoxic stress, cell cycle arrest, autophagy, apoptosis, and cell senescence [38–42]. In addition to growth inhibition, MLN4924 has been reported to suppress cell migration in lung and urothelial carcinomas and to reduce cancer metastasis in animals [43, 44]. However, Zhou et al. recently claimed that MLN4924 at a low concentration (30–100 nM) stimulated cancer cell proliferation, sphere formation, and tumorigenesis [45]. Consistent with this report, we observed that even at moderate nanomolar concentrations (250 and 500 nM) MLN4924 accelerated cell migration at least in two cancer cell-lines. MLN4924 may affect cancer cell migration in different ways depending on cell contexts.

## Conclusions

Our results suggest that the neddylation of caveolin-1 interferes with the Src-mediated Y14-phosphorylation of caveolin-1 and by doing so suppresses cell migration. This study may provide a better understanding of the mechanism regulating cell movement. Surprisingly, at least in our experimental settings, an emerging anticancer drug MLN4924 was shown to enhance migration of prostate cancer and glioblastoma cells. Therefore, the possibility that MLN4924 could aggravate cancer progress under some circumstances should be carefully checked before this drug will come to the market.

## Additional files

Additional file 1: MLN4924 selectively inhibits NEDD8 activating enzyme (NAE) in the range of 0.25–0.5 μM. To examine the possible inhibition of the related enzymes ubiquitin-activating enzyme (UAE) and SUMO-activating enzyme (SAE), PC3 and U373MG cells were treated with 0.25 μM and 0.5 μM of MLN4924 for 24 h, respectively. Total modified forms of proteins by NEDD8, ubiquitin, and SUMO in cell lysates were analyzed by Western blotting with indicated antibodies. The doses of MLN4924 applied in our experiments (PC3: 0.25 μM, U373MG: 0.5 μM) inhibited neddylation without affecting ubiquitination and sumoylation. (PPTX 73 kb)

Additional file 2: N-terminal myc tagged caveolin-1 failed to be covalently conjugated with NEDD8. Ni-NTA-binding assay was performed in HEK293T cells co-expressing His-NEDD8 and myc-caveolin-1. Neddylated proteins were pulled down with Ni-NTA beads under a denaturing condition, and subjected to Western blotting with the indicated antibodies. (PPTX 18756 kb)

Additional file 3: Phosphorylated caveolin-1 is essential for MLN4924-induced cell migration. Scratch-based wound healing assays were performed for 24 h in PC3 (A) and U373MG (B) cells which were depleted of caveolin-1 using siRNA (#1 and #2, respectively) and si-control in the presence of MLN4924 (0.25 μM and 0.5 μM) or DMSO (top). The migration areas were calculated using ImageJ at just below. Proteins in cells lysates were analyzed by Western blotting (middle). The efficiency of the caveolin-1 knock-down and magnitude of the phosphorylation of caveolin-1 was quantified based upon the relative level of β-tubulin (bottom). Each bar represents the means + standard deviation of results from three independent experiments. * denotes *P* < 0.05 and n, s, does *P* > 0.05 between the indicated groups. Scale bar = 200 μm. (PPTX 12560 kb)

Additional file 4: Neddylation inhibition enhances the Src-mediated phosphorylation of caveolin-1. Scratch-based wound healing assays were performed for 24 h in PC3 (A) and U373MG (B) cells which were depleted of NEDD8 using siRNA #2 and si-control in the absence or presence 10 μM PP2 (top). The migration areas were calculated using ImageJ at just below. Proteins in cells lysates were analyzed by Western blotting (middle). The level of the phosphorylation of caveolin-1 was quantified based upon the relative level of β-tubulin (bottom). Each bar represents the means + standard deviation of results from three independent experiments. * denotes *P* < 0.05 between the indicated groups. Scale bar = 200 μm. (PPTX 21157 kb)

Additional file 5: PP2 can affect multiple cellular responses involving migration. A. Scratch-based wound healing assays were performed for 24 h in the vehicle (A) and si-control (C) of PC3 and U373MG cells which were treated with 10 μM PP2 (top). The migration areas were calculated using ImageJ at just below. Protein levels in cells lysates were analyzed by Western blotting. The PP2 mediated inhibition of the phosphorylation of caveolin-1 was quantified based upon the relative level of β-tubulin (bottom). Transwell migration assays were performed in the vehicle (B) and si-control (D) of PC3 and U373MG cells treated with 10 μM PP2 (left), and migrated cells were counted (right). Each bar represents the means + standard deviation of results from three independent experiments. * denotes P < 0.05 between the indicated groups. Scale bar = 200 μm. (PPTX 24837 kb)

## Abbreviations

APPBP1: Amyloid beta precursor protein-binding protein 1
Cav1: Caveolin-1
CRL: Cullin ring ligases
FAK: Focal adhesion kinase
Grb7: Growth factor receptor-bound protein 7
HIF-1α: Hypoxia-inducible factor 1-alpha
Mdm2: Mouse double minute 2
NAE: NEDD8-activating enzyme
NEDD8: Neural precursor cell expressed, developmentally downregulated 8
RING: Really interesting new gene
UBA3: Ubiquitin-like modifier activating enzyme 3
UBC12: NEDD8-conjugating enzyme 12
