# Supplementary figures and images for: Inhibition of neddylation facilitates cell migration through enhanced phosphorylation of caveolin-1 in PC3 and U373MG cells
Source: BMC Cancer. 2018 Jan 5;18:30. doi: 10.1186/s12885-017-3942-9 (PMC5755266; doi:10.1186/s12885-017-3942-9)

## Slide 1
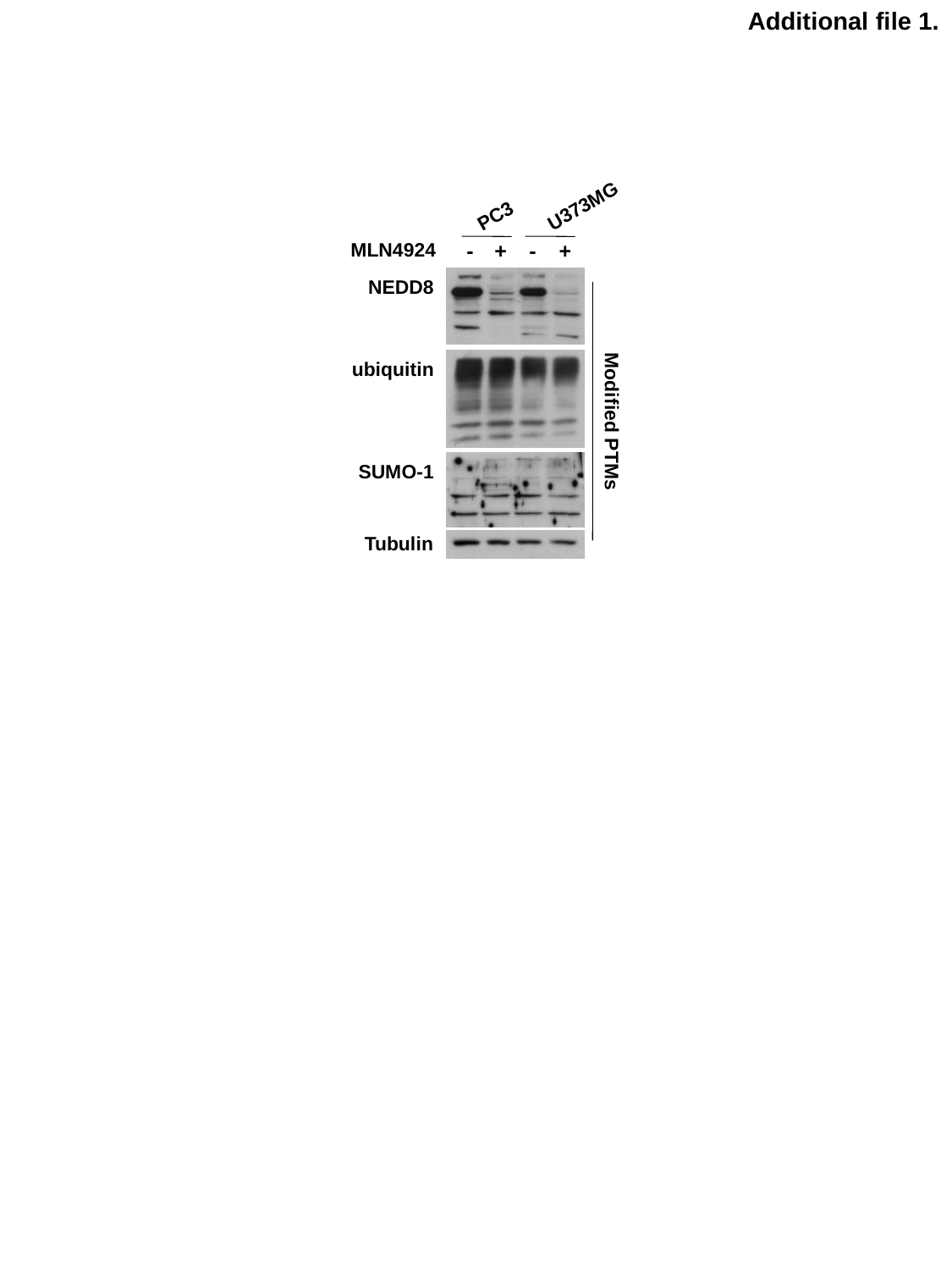

Additional file 1.
U373MG
PC3
| - | + | - | + |
| --- | --- | --- | --- |
| MLN4924 |
| --- |
NEDD8
ubiquitin
Modified PTMs
SUMO-1
Tubulin

Supplement: Supplementary file 1 — MLN4924 selectively inhibits NEDD8 activating enzyme (NAE) in the range of 0.25–0.5 μM. To examine the possible inhibition of the related enzymes ubiquitin-activating enzyme (UAE) and SUMO-activating enzyme (SAE), PC3 and U373MG cells were treated with 0.25 μM and 0.5 μM of MLN4924 for 24 h, respectively. Total modified forms of proteins by NEDD8, ubiquitin, and SUMO in cell lysates were analyzed by Western blotting with indicated antibodies. The doses of MLN4924 applied in our experiments (PC3: 0.25 μM, U373MG: 0.5 μM) inhibited neddylation without affecting ubiquitination and sumoylation. (PPTX 73 kb) [file 12885_2017_3942_MOESM1_ESM.pptx]

## Slide 1
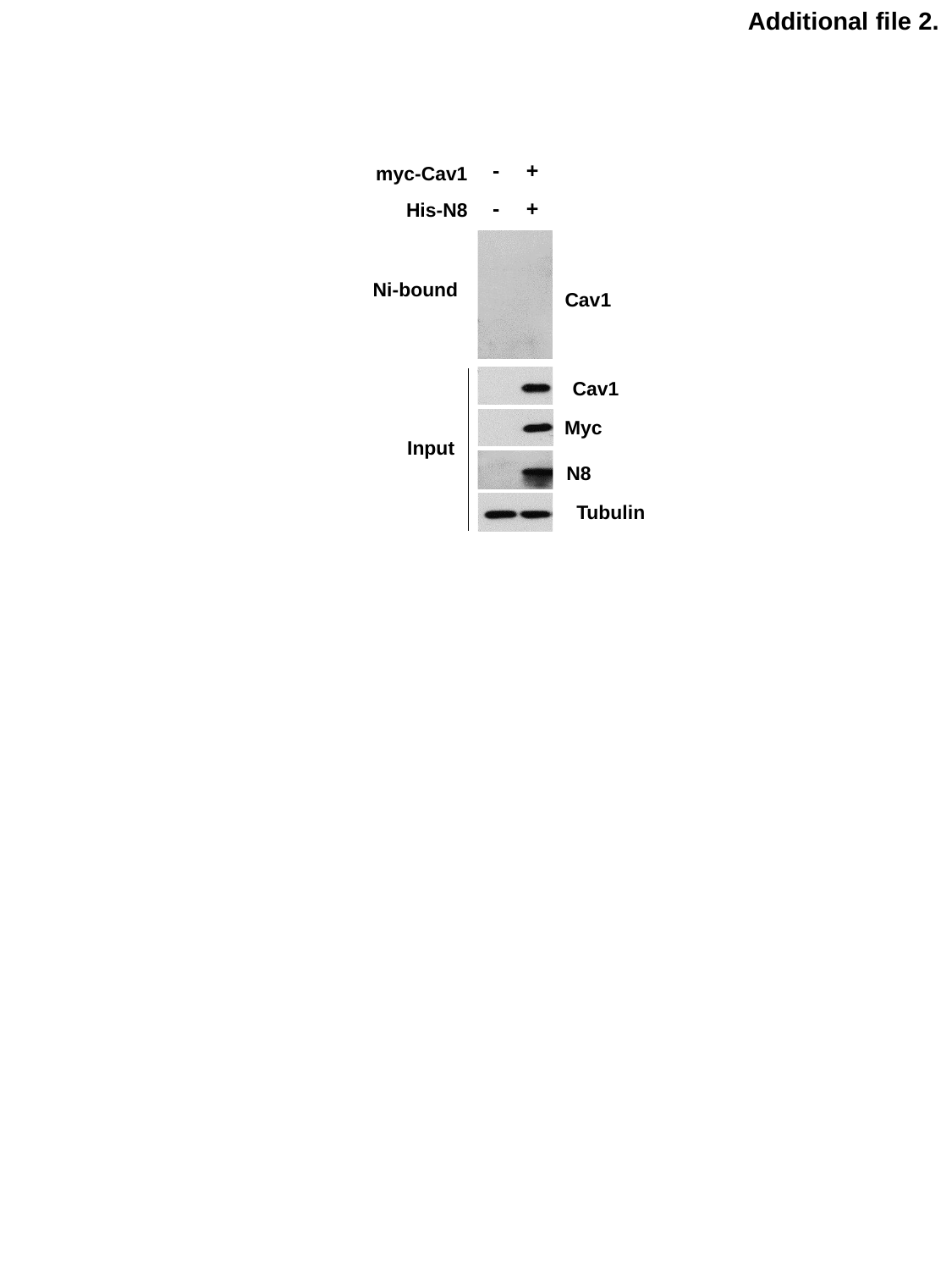

Additional file 2.
| - | + | |
| --- | --- | --- |
| - | + | |
| myc-Cav1 |
| --- |
| His-N8 |
Ni-bound
Cav1
Cav1
Myc
Input
N8
Tubulin

Supplement: Supplementary file 2 — N-terminal myc tagged caveolin-1 failed to be covalently conjugated with NEDD8. Ni-NTA-binding assay was performed in HEK293T cells co-expressing His-NEDD8 and myc-caveolin-1. Neddylated proteins were pulled down with Ni-NTA beads under a denaturing condition, and subjected to Western blotting with the indicated antibodies. (PPTX 18756 kb) [file 12885_2017_3942_MOESM2_ESM.pptx]
