# Supplementary material for: Inhibition of neddylation facilitates cell migration through enhanced phosphorylation of caveolin-1 in PC3 and U373MG cells
Source: BMC Cancer. 2018 Jan 5;18:30. doi: 10.1186/s12885-017-3942-9 (PMC5755266; doi:10.1186/s12885-017-3942-9)
Supplement: Supplementary file 3 — Phosphorylated caveolin-1 is essential for MLN4924-induced cell migration. Scratch-based wound healing assays were performed for 24 h in PC3 (A) and U373MG (B) cells which were depleted of caveolin-1 using siRNA (#1 and #2, respectively) and si-control in the presence of MLN4924 (0.25 μM and 0.5 μM) or DMSO (top). The migration areas were calculated using ImageJ at just below. Proteins in cells lysates were analyzed by Western blotting (middle). The efficiency of the caveolin-1 knock-down and magnitude of the phosphorylation of caveolin-1 was quantified based upon the relative level of β-tubulin (bottom). Each bar represents the means + standard deviation of results from three independent experiments. * denotes P < 0.05 and n, s, does P > 0.05 between the indicated groups. Scale bar = 200 μm. (PPTX 12560 kb) [file 12885_2017_3942_MOESM3_ESM.pptx]

## Slide 1
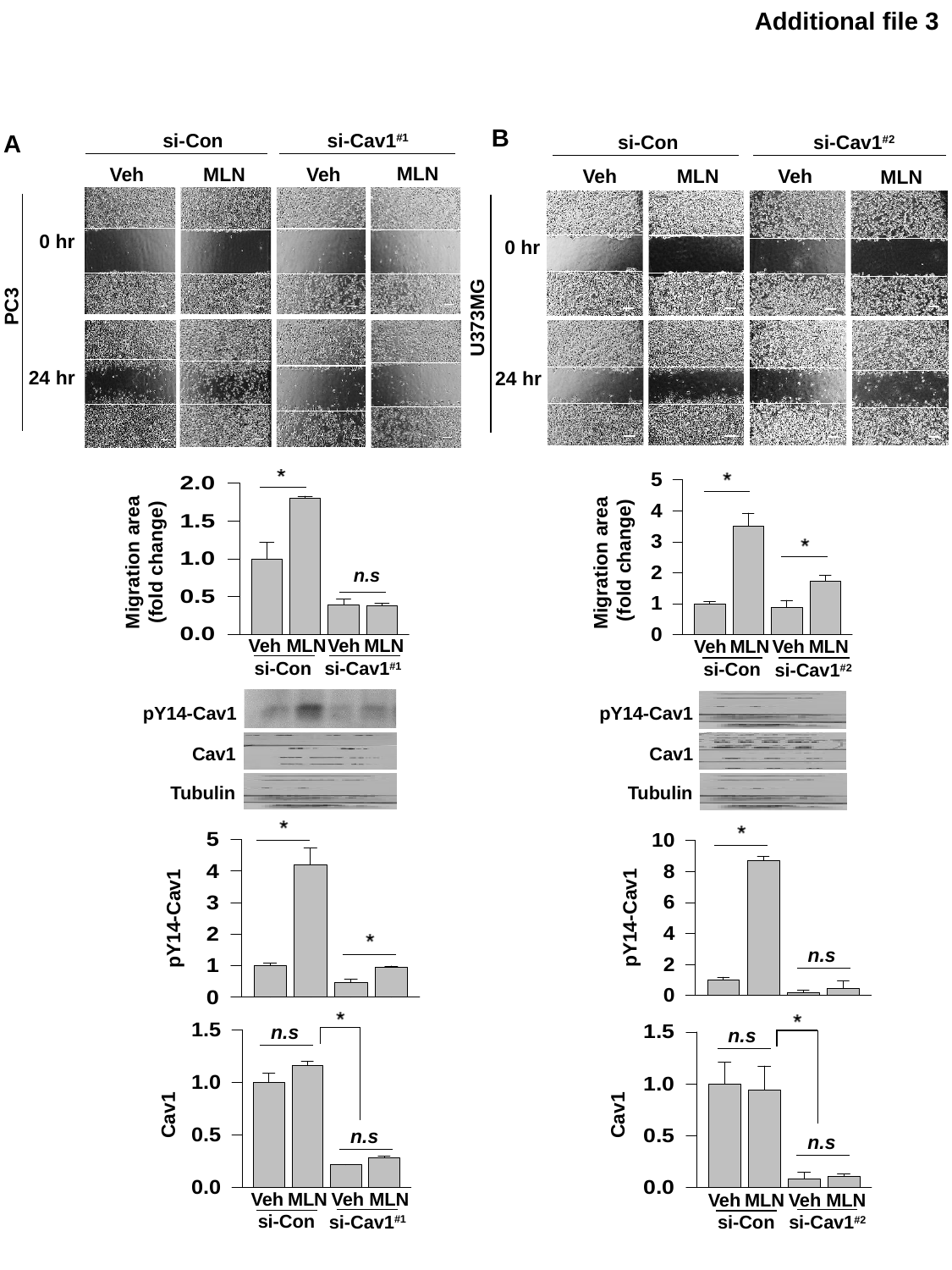

Additional file 3
B
si-Con
si-Cav1#2
Veh
MLN
Veh
MLN
0 hr
U373MG
24 hr
A
si-Con
si-Cav1#1
MLN
Veh
MLN
Veh
0 hr
PC3
24 hr
Migration area
(fold change)
n.s
Veh
MLN
Veh
 MLN
 si-Con
si-Cav1#1
Migration area
 (fold change)
Veh
MLN
Veh
 MLN
 si-Con
si-Cav1#2
pY14-Cav1
Cav1
Tubulin
pY14-Cav1
Cav1
Tubulin
pY14-Cav1
pY14-Cav1
n.s
Cav1
Veh
MLN
Veh
 MLN
 si-Con
si-Cav1#1
n.s
n.s
Cav1
Veh
MLN
Veh
 MLN
 si-Con
si-Cav1#2
n.s
n.s
