# Supplementary material for: Inhibition of neddylation facilitates cell migration through enhanced phosphorylation of caveolin-1 in PC3 and U373MG cells
Source: BMC Cancer. 2018 Jan 5;18:30. doi: 10.1186/s12885-017-3942-9 (PMC5755266; doi:10.1186/s12885-017-3942-9)
Supplement: Supplementary file 4 — Neddylation inhibition enhances the Src-mediated phosphorylation of caveolin-1. Scratch-based wound healing assays were performed for 24 h in PC3 (A) and U373MG (B) cells which were depleted of NEDD8 using siRNA #2 and si-control in the absence or presence 10 μM PP2 (top). The migration areas were calculated using ImageJ at just below. Proteins in cells lysates were analyzed by Western blotting (middle). The level of the phosphorylation of caveolin-1 was quantified based upon the relative level of β-tubulin (bottom). Each bar represents the means + standard deviation of results from three independent experiments. * denotes P < 0.05 between the indicated groups. Scale bar = 200 μm. (PPTX 21157 kb) [file 12885_2017_3942_MOESM4_ESM.pptx]

## Slide 1
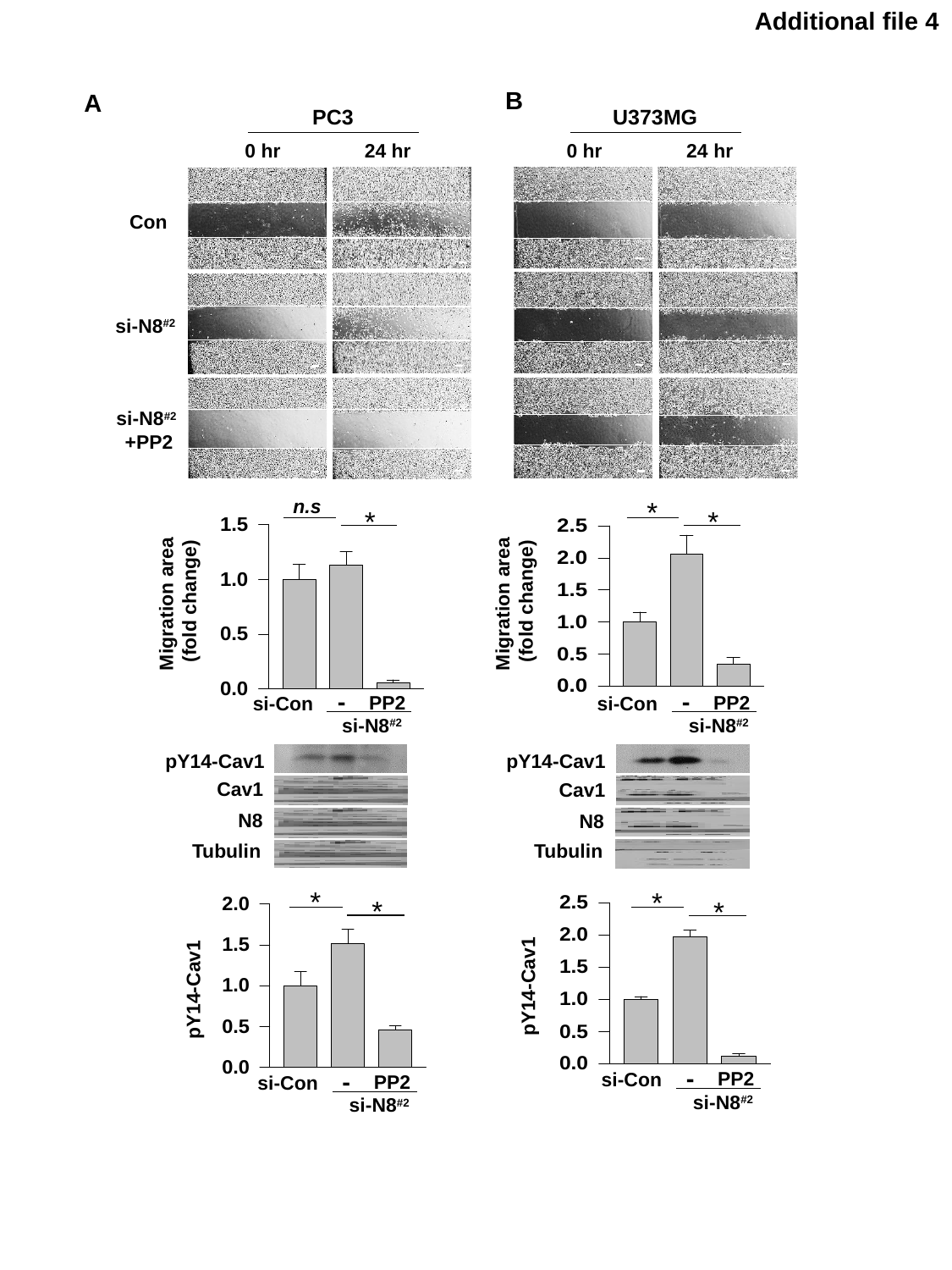

Additional file 4
B
A
PC3
0 hr
24 hr
Con
si-N8#2
si-N8#2
 +PP2
U373MG
0 hr
24 hr
*
*
-
PP2
si-Con
si-N8#2
Migration area
 (fold change)
n.s
*
Migration area
 (fold change)
-
PP2
si-Con
si-N8#2
pY14-Cav1
Cav1
N8
Tubulin
pY14-Cav1
Cav1
N8
Tubulin
*
*
*
pY14-Cav1
*
pY14-Cav1
-
-
PP2
si-Con
PP2
si-Con
si-N8#2
si-N8#2
