# Supplementary material for: Inhibition of neddylation facilitates cell migration through enhanced phosphorylation of caveolin-1 in PC3 and U373MG cells
Source: BMC Cancer. 2018 Jan 5;18:30. doi: 10.1186/s12885-017-3942-9 (PMC5755266; doi:10.1186/s12885-017-3942-9)
Supplement: Supplementary file 5 — PP2 can affect multiple cellular responses involving migration. A. Scratch-based wound healing assays were performed for 24 h in the vehicle (A) and si-control (C) of PC3 and U373MG cells which were treated with 10 μM PP2 (top). The migration areas were calculated using ImageJ at just below. Protein levels in cells lysates were analyzed by Western blotting. The PP2 mediated inhibition of the phosphorylation of caveolin-1 was quantified based upon the relative level of β-tubulin (bottom). Transwell migration assays were performed in the vehicle (B) and si-control (D) of PC3 and U373MG cells treated with 10 μM PP2 (left), and migrated cells were counted (right). Each bar represents the means + standard deviation of results from three independent experiments. * denotes P < 0.05 between the indicated groups. Scale bar = 200 μm. (PPTX 24837 kb) [file 12885_2017_3942_MOESM5_ESM.pptx]

## Slide 1
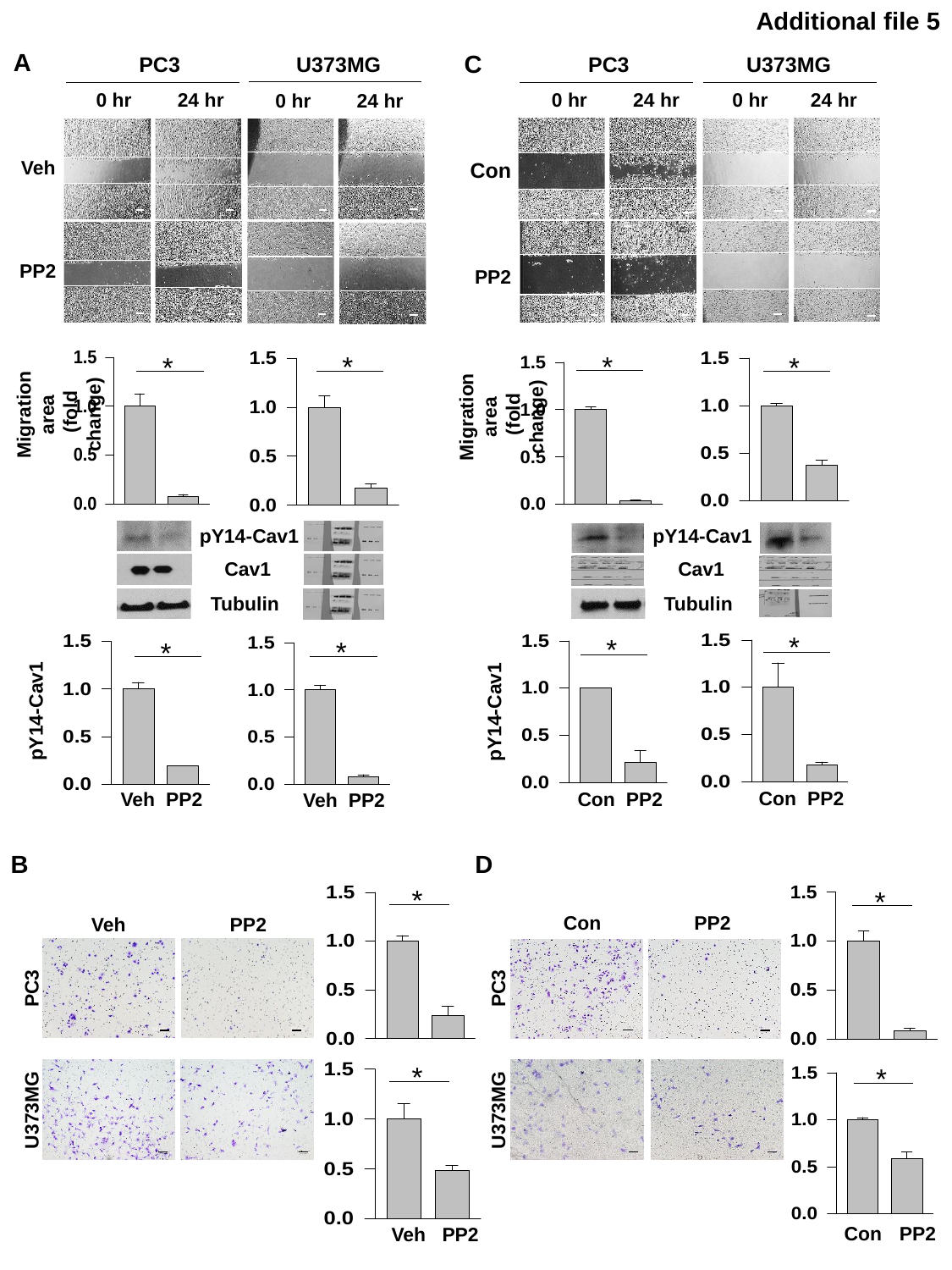

Additional file 5
A
U373MG
PC3
0 hr
24 hr
0 hr
24 hr
Veh
PP2
C
PC3
U373MG
0 hr
24 hr
0 hr
24 hr
Con
PP2
*
Migration area
 (fold change)
*
*
*
Migration area
 (fold change)
pY14-Cav1
Cav1
Tubulin
pY14-Cav1
Cav1
Tubulin
pY14-Cav1
*
Veh PP2
pY14-Cav1
*
Con PP2
*
Con PP2
*
Veh PP2
B
D
Con PP2
PC3
*
*
Veh PP2
PC3
Veh
PP2
*
*
Con
PP2
U373MG
U373MG
